# Effect of education and attitude on health professionals’ knowledge on prenatal screening

**DOI:** 10.18332/ejm/126626

**Published:** 2020-09-22

**Authors:** Charlotte H. Jansen, Jana M. de Vries, Melanie Engels, Karline van de Kamp, Rosalinde J. Snijders, Linda Martin, Lidewij Henneman, Eva Pajkrt

**Affiliations:** 1 Department of Obstetrics and Gynaecology, Amsterdam Reproduction and Development Research Institute, Amsterdam UMC, University of Amsterdam,Amsterdam, the Netherlands; 2 EchoXpert, Prenatal Ultrasound and Training Center, Amsterdam,the Netherlands; 3 Midwifery Science, Amsterdam Public Health Research Institute, Amsterdam UMC, Vrije Universiteit Amsterdam, Amsterdam, the Netherlands; 4 Department of Clinical Genetics, Amsterdam Reproduction and Development Research Institute, Amsterdam UMC, Vrije Universiteit Amsterdam, Amsterdam, the Netherlands

**Keywords:** knowledge, screening, attitude, counselling

## Abstract

**INTRODUCTION:**

Ongoing developments in prenatal anomaly screening necessitate continuous updating of counsellors’ knowledge. We explored the effect of a refresher counselling course on participants’ knowledge of prenatal screening.

**METHODS:**

We investigated the association between knowledge and counsellors’ working experience. Also, the association between knowledge and counsellors’ attitude towards prenatal screening was determined. All counsellors in the North-West region of the Netherlands were invited to attend a refresher counselling course and fill in both a pre-course and a post-course questionnaire. The participants consisted of midwifes, sonographers and gynaecologists. A 55-item questionnaire assessed pre-course (T0) and post-course (T1) knowledge. At T0, counsellors’ attitude towards the prenatal screening program was assessed and its association with knowledge analysed.

**RESULTS:**

Of 387 counsellors, 68 (18%) attended the course and completed both questionnaires. Knowledge increased significantly from 77.7% to 84.6% (p<0.01). Scores were lowest regarding congenital heart diseases. Participants with ultrasound experience scored higher on T0, but improvement was seen in participants with and without ultrasound experience. Participants with a positive attitude towards a free-of-charge first trimester combined test had higher knowledge scores than participants with a negative attitude (62% vs 46%; p=0.002).

**CONCLUSIONS:**

A refresher course improved counsellors’ knowledge on prenatal screening. Ultrasound experience and a positive attitude towards free screening may be associated with higher knowledge levels. Participating in a mandatory refresher counselling course is useful for the continuous improvement of healthcare practitioners’ knowledge. More research on the effect of knowledge and attitude on the quality of prenatal screening is necessary.

## INTRODUCTION

As part of routine prenatal care, many countries offer prenatal screening for aneuploidies such as Down syndrome and for foetal structural anomalies^1^. The ability for women to make an informed decision to pursue or decline prenatal screening is based on the right to make autonomous reproductive choices ^2^. Sufficient knowledge and understanding of all options combined with the women’s values are necessary to make an informed choice and therefore women are counselled by healthcare providers. However, women do not always understand the implications of the prenatal tests, nor do they feel that they have been well informed by counsellors^3,4^. Fast developments in screening possibilities over the past decade resulted in many changes in first and second trimester screening. This necessitates frequent education to update counsellors’ knowledge.

In the Netherlands, all pregnant women are asked whether they wish to receive information on prenatal screening for foetal anomaly. If they agree, a trained and certified counsellor, mostly a primary care midwife, informs the women about the options of prenatal screening. From 2007 onwards, first trimester screening with the combined test (CT) and second trimester screening with the foetal anomaly scan (FAS) are offered. In April 2014, the option of non-invasive prenatal testing (NIPT) was added for women with increased risk for trisomy 21, 18 or 13 based on CT or medical history (TRIDENT-1 study)^5^. Since April 2017, NIPT has been available for all pregnant women irrespective of their background risk (TRIDENT-2 study)^6^. In the Netherlands, a counselling license is acquired with finishing midwifery education and an online test on prenatal screening or after a counselling training programme and the online test. In order to help future parents to make well-informed decisions, healthcare providers should have sufficient knowledge of prenatal screening and continuously educate themselves to optimize their counselling^7^. Furthermore, the principle of non-directiveness is important; the counsellor refrains from offering his ow beliefs and is value neutral. This promotes the principle of autonomy of women since an autonomous, informed decision is one that is consistent with the decision makers values^8^. Although counsellors generally strive to value neutrality, in practice this principle may not always be adhered to. Several studies indicate that personal attitudes of healthcare professionals influence the choices parents make concerning prenatal screening^9-11^. However, van den Berg et al.^12^ showed no effect of Dutch counsellors’ attitudes on the parents’ attitudes and decisions about prenatal screening. Counsellors’ attitude might also influence knowledge, since earlier research demonstrated that more positive attitudes towards prenatal screening for Down syndrome was associated with higher knowledge scores on NIPT^13^. Moreover, differences in attitudes towards prenatal screening with NIPT were observed between different professions, e.g. with and without ultrasound experience^14^.

To investigate whether a refresher counselling course should be mandatory, its usefulness should be tested. Therefore, we investigated the effect of a refresher course on counsellors’ knowledge about prenatal screening. Also, we determined the association between knowledge and counsellors’ ultrasound and working experience and the association between knowledge and counsellors’ attitude towards prenatal screening.

## METHODS

### Design, participants and procedure

This study was performed in March 2015 by the Regional Prenatal Centers, the Stichting Prenatale Screening Amsterdam en Omstreken (SPSAO) and Regionaal Centrum Prenatale Screening Noord-Holland (RCPSNH) in the North-West part of the Netherlands. All 564 counsellors, registered with one of the centers for counselling, were personally invited to attend a non-mandatory refresher course on prenatal screening. A general invitation was sent to hospital obstetric departments and primary midwifery practices. There was a separate invitation to fill in the questionnaire. Both invitations were sent by email. Counsellors in the Netherlands are midwives, sonographers, gynaecologists, fertility doctors or specialized nurses.

During the one-day course, developments in prenatal screening were summarized and results of the TRIDENT-1 study were presented^5,15^. Experiences of pregnant women with NIPT in the TRIDENT-1 study were presented and the added value of a first trimester ultrasound exam was addressed^16^. The lectures were followed by knowledge quizzes and group discussions. Participants were shown videos of healthcare professionals and parents simulating prenatal counselling. In a group discussion, the quality of the counselling method in the videos was evaluated.

Irrespective of their intent to attend the course, all counsellors were asked to fill in an online questionnaire before the course (T0) to assess attitude and knowledge of prenatal screening. Two weeks after the course (T1), attendants were again asked to fill in the same questionnaire to assess knowledge. To increase response rates, a reminder email was sent after 20 days. An expert panel specialized in prenatal counselling and/or prenatal diagnostic tests designed the questionnaire and course. The questions are shown in the Supplementary file. All participants were certified for counselling at the time of filling in the first the questionnaire. The questionnaires were non-anonymous, so course attendance could be linked to the participants’ test scores, but during analysis participants were pseudonymised. The participating healthcare professionals were informed that the questionnaires were to be used for research and that results could be used for publication. Medical ethics committee approval was acquired (W19_312 #19.370).

### Data collection

In total, 598 counsellors were invited to complete the 55-item pre-course questionnaire (T0). The pre-course questionnaire was divided into three subsections. In the first section participants were asked to report their professional background and the years of counselling experience. Participants were also asked to report whether or not they performed ultrasound exams. The second section assessed knowledge about issues relevant to prenatal screening, aneuploidies and structural anomalies. Questions were divided in subcategories according to subject with categories as follows: Combined test (n=12; 24%), NIPT (n=11; 22%), the foetal anomaly scan (FAS) (n=12; 24%), aneuploidies (n=17; 31%) and structural anomalies (n=6; 12%). The latter two were subdivided into questions about trisomy 21 (n=12; 24%), trisomy 18 (n=2; 4%) and trisomy 13 (n=3; 5%) as aneuploidies and spina bifida (n=2; 4%), and congenital heart diseases (n=4; 7%) as structural anomalies.

The last section assessed statements regarding personal attitude towards prenatal anomaly screening. The participants could either agree or disagree with each statement or give neutral response. The statements were: 1) Screening for Down syndrome should not be available, 2) The foetal anomaly scan (FAS) has been offered too easily, 3) The combined test (CT) should be free-of-charge for everyone who wants to have the test, and 4) The NIPT should be available for everyone who wants to have the test.

After the course at T1, the participants completed a questionnaire that only addressed knowledge.

### Data analysis

The main outcome was difference in knowledge after the refresher course in the total group, and in healthcare professionals with and without ultrasound experience. The percent correct answers of the participants that responded both pre-course (T0) and post-course (T1) were calculated. We compared the difference in test score at T0 and T1 for the group with and without ultrasound experience and for counsellors who recently started (0–5 years active as a counsellor) and experienced counsellors (≥6 years). A paired t-test was used to compare test scores.

Subsequently, we calculated the difference in score per subgroup for CT, NIPT, FAS, aneuploidies, and structural anomalies. For comparison, we used test scores ≥75% correct, since the groups were too small to use the percentages. Again, we compared between participants with and without ultrasound experience. Finally, we analysed the association between overall knowledge at T0 and attitude about prenatal screening. The attitude questions that participants answered during the pre-course questionnaire were compared to the knowledge score of the pre-course test using ≥75% correct answers. For both analysis we used a Pearson’s chi-squared test.

All test scores were noted as mean percentage of correctly given answers with standard deviation (SD). P-values and confidence intervals were calculated, and p=0.01 was considered as the threshold for statistical significance. All data were collected and analysed using IBM SPSS for Windows, version 23.

## RESULTS

**Figure 1:**
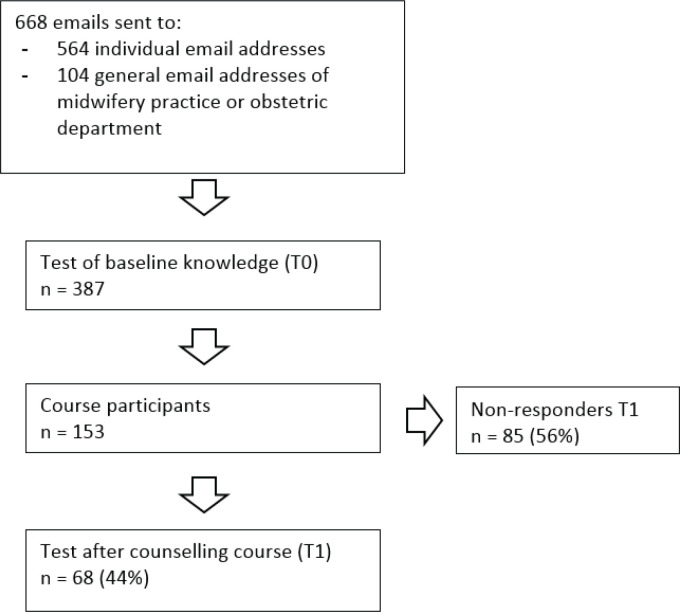
Flowchart of participant recruitment and course and test aprticipation.

The baseline questionnaire T0 was completed by 387 counsellors. Of those, 153 (40%) participated in the refresher course and 68 (18%) completed the T1 questionnaire (Table 1 and Figure 1).

**Table 1:** Baseline characteristics of participants at T0 and of participants who completed the course and the pre-course (T0) and post-course (T1) test

*Characteristics*	*Total (N=387) n (%)*	*T0 + Course participation + T1 (N=68) n (%)*
**Profession**		
Midwife	313 (81)	50 (74)
Sonographer	31 (8)	15 (22)
Gynaecologist	34 (9)	3 (4)
Other^a^	9 (2)	0 (-)
**Ultrasonography experience**		
Yes	280 (72)	31 (46)
No	107 (28)	37 (54)
**Years of experience as counsellor**		
0–5	157 (41)	27 (40)
≥6	230 (59)	41 (60)
**Contract with Regional Prenatal Centre**		
SPSAO	146 (38)	24 (35)
RCPSNH	148 (38)	28 (41)
Both	50 (13)	6 (9)
Other^b^	39 (10)	10 (15)

a In vitro fertilization doctors, nurse-practitioners, midwife students.

b Not active in Amsterdam region. SPSAO: Stichting Prenatale Screening Amsterdam en Omstreken, RCPSNH: Stichting Regionaal Centrum Prenatale Screening NoordHolland.

### Pre-course and post-course knowledge scores

The percentage of correct answers on the knowledge questions of the 68 attendants that completed both the precourse and post-course questionnaire significantly improved after the course (77.7% vs 84.6%, respectively; 95% CI: -8.5 – -5.4; p<0.01) (Table 2). Improvement was seen both in participants with ultrasound experience and in participants without ultrasound experience (Table 2). At T0, before the refresher course, participants with ultrasound experience had a higher mean percentage of correct answers compared to participants without ultrasound experience (81.1% vs 74.7%, respectively; 95% CI: 3.2–9.7; p<0.001). At T1, after the refresher course, the mean percentage of correct answers did not differ significantly between participants with and without ultrasound experience (86.3% vs 83.1%, respectively; 95% CI: 0.2–6.2; p=0.038). The percentage of correct answers did not differ at T0 between both groups with different years of experience, but improved significantly in both groups after the course (Table 2).

**Table 2:** Comparing counsellors’ knowledge scores between T0 and T1

	*Per cent correct answers at T0 Mean (SD)*	*Per cent correct answers at T1 Mean (SD)*	*p*	*(95% CI)^b^*
**Total^a^** (N=68)	77.7 (7.3)	84.6 (6.4)	<0.01	(-8.5 – -5.4)
**Ultrasonography experience**				
Yes (n=31)	81.1 (6.9)	86.3 (5.7)	<0.01	(-7.3 – -3.0)
No (n=37)	74.7 (6.2)	83.1 (6.6)	<0.01	(-10.6 – -6.2)
**Years of experience as counsellor**				
0–5 (n=27)	77.4 (5.5)	84.1 (6.0)	<0.01	(-9.0 – -4.4)
≥6 (n=41)	107 (28.0)	37 (54.0)	<0.01	(-9.3 – -4.8)

a Questionnaire T0 + course participation + questionnaire T1.

b Difference between T0 and T1 with significance at p<0.01 (95% CI).

### Pre-course knowledge on questionnaire subcategories

At T0 a relatively large proportion of counsellors had ≥75% correct answers on questions concerning CT (n=306; 81%) and NIPT (n=299; 79%) compared to scores on FAS questions (n=161; 43%) (Table 3). The questions most often answered incorrectly concerned the next steps to be taken in case of an incomplete foetal anomaly scan at 20 weeks and questions concerning congenital heart disease.

**Table 3:** Comparing counsellors’ knowledge (≥75% correct answers) on different subcategories at T0, for counsellors with and without ultrasonography (USG) experience

*Questionnaire subcategories (number of questions)*	*Total number of participants (N=377) n (%)*	*Participants with USG experience(N=104) n (%)*	*Participants without USG experience(N=273) n (%)*	*p^a^*
**FAS** (n=12)	161 (43)	48 (46)	113 (41)	0.40
**CT** (n=12)	306 (81)	98 (94)	208 (76)	<0.001
**NIPT** (n=11)	299 (79)	90 (87)	209 (77)	0.03
**Aneuploidies**	244 (65)	83 (80)	161 (59)	<0.001
Trisomy 21 (n=12)	203 (54)	72 (69)	131 (48)	<0.001
Trisomy 13 (n=2)	304 (81)	89 (86)	215 (79)	0.13
Trisomy 18 (n=3)	295 (78)	86 (83)	209 (77)	0.20
**Structural anomalies**	250 (66)	76 (73)	174 (64)	0.09
Spina bifida (n=2)	292 (78)	88 (85)	204 (75)	0.04
Congenital heart disease (n=4)	103 (27)	27 (26)	76 (28)	0.72

a Difference between practitioners with and without ultrasonography experience with significance at p<0.01.

FAS: foetal anomaly scan. CT: combined test. NIPT: noninvasive prenatal test. USG: ultrasonography.

These questions were correctly answered by only 84 (23%) and 103 (27%) of the participants, respectively.

Questions concerning aneuploidies (n=17) and structural anomalies (n=6) were correctly answered (≥75% correct answers) by 256 (66%) and 246 (64%) participants, respectively.

When comparing counsellors with ultrasound experience and without ultrasound experience, the group with ultrasound experience had a higher knowledge score on CT and chromosome anomalies (p<0.001).

### Counsellors’ attitude and knowledge

Participants with a positive attitude towards offering the CT for free had a significant higher test score than participants with a negative attitude (62% vs 46%, respectively; χ^2^(1)=9.48; p=0.002). For the other attitude statements there was no difference in knowledge scores (Table 4). There was no difference in test score in the group who responded positively versus the participants that refrained from answering (62% vs 55%, χ^2^(1)=0.52, p=0.5; and 46% vs 55%, χ^2^(1)=0.56, p=0.5).

**Table 4:** Association between counsellors’ attitudes towards prenatal screening (statements) and knowledge (≥75% correct answers)

*Statements*	*Total number of participants (N=377) n (%)*	*Number of participants with ≥75% correct answers at T0(N=209) n (%)*
**Screening for Down syndrome should not be available**		
Agree	6 (2)	3 (50)
Disagree	356 (94)	198 (56)
Would rather not answer	15 (4)	8 (53)
**The FAS is offered too easily**		
Agree	95 (25)	43 (45)
Disagree	274 (73)	161 (59)
Would rather not answer	8 (2)	5 (63)
**The CT should be free-ofcharge for everyone who wants to have the test**		
Agree	205 (54)	128 (62)^a^
Disagree	150 (40)	69 (46)^a^
Would rather not answer	22 (6)	12 (55)
**The NIPT should be available for everyone who wants to have the test**		
Agree	258 (68)	142 (55)
Disagree	102 (27)	56 (55)
Would rather not answer	17 (5)	11 (65)

a Significant difference in knowledge score.

FAS: foetal anomaly scan. CT: combined test. NIPT: non-invasive prenatal test.

## DISCUSSION

Our findings show that providing a refresher course on prenatal anomaly screening is effective for counsellors with and without ultrasound experience. Both groups show a similar level of knowledge after a counselling course. However, before the course, counsellors with ultrasound experience had a higher level of knowledge on prenatal screening than counsellors without ultrasound experience. Before the course, participants with ultrasound experience scored higher compared to participants without ultrasound experience on questions about the CT and chromosome anomalies. Length of working experience does not influence the counsellors’ knowledge score before or after a refresher course. Counsellors with a positive attitude towards first trimester screening being free-of-charge have higher knowledge scores.

The finding that a refresher course is an effective way to improve counsellors’ knowledge on prenatal anomaly screening is in agreement with earlier research^17^. Up-to-date knowledge on screening options, conditionally increases the quality of counselling, although further research is needed to assess whether improved knowledge of the counsellor also improves the information provided to women and their partners. The finding that knowledge of counsellors with ultrasound experience is superior to that of counsellors who do not provide ultrasound assessments may be associated with the general higher exposure to prenatal anomalies in the former group. Significant differences in knowledge scores have previously been reported between different clinical work areas. Oxenford et al.^17^ showed that foetal medicine midwives scored significantly higher than student midwives, general midwives and community midwives. Similar to our findings, the post-training follow-up assessment did no longer reveal any difference in test scores.

We found that counsellors scored differently on various subjects. Before the course, just over half of the participants had ≥75% correct answers. The literature indicates that counsellors’ knowledge of Down syndrome needs to be improved to ensure that they can provide women with information to make informed decisions^18-20^. Overall, knowledge of first trimester screening was better than knowledge on the second trimester anomaly scan, mainly due to the lack of knowledge about congenital heart diseases in the second trimester. Prenatal detection of congenital heart diseases which require intervention is important since planned delivery and appropriate postnatal care in those cases improve the postpartum outcome^21,22^. In our study, years of counselling experience was not associated with knowledge. These findings are consistent with a study by Ternby et al.^18^ showing no significant difference in knowledge about Down syndrome between midwife counsellors who had worked >10 years in the field compared to midwife counsellors who had worked ≤10 years^18^. However, in their study, midwives with more experience in practice did feel more secure about their knowledge compared to less experienced midwives. Finally, we found that participants’ positive attitude towards the statement ‘the combined test should be offered free-of-charge to everyone who wants to have the test’, had a higher knowledge score than participants with a negative attitude towards this statement. In the Netherlands, the cost of first trimester screening has been an issue of debate, as some have argued that this can be used to motivate women to think more thoroughly about their decision to screen, whereas others see this as a barrier in the access to screening^23^. A positive association between counsellors’ general attitude toward prenatal screening for Down syndrome and knowledge was shown in a national survey study among Dutch counsellors^13^. Since we evaluated attitudes only before the counselling course (T0) we can only investigate the association rather than the causality between counsellors’ attitude and knowledge. On the one hand, it can be argued that healthcare professionals may have a negative attitude towards a statement because they know less about the specific topic. On the other hand, it might be that professionals who support first-trimester prenatal screening are more interested in the topic and thus know more about it.

The percentage of correct answers of the participants was 77.7% pre-course and improved to 86.4% postcourse. Although the course is shown to be helpful to improve knowledge, it is still unclear if this improvement is long-term. Earlier research showed that the effect of a training session is at least maintained for a month^17^. At the moment of this study, a refresher counselling course was not mandatory, however, since 2017 in the Netherlands, attending a refresher course is mandatory every two years. The reason for instating the mandatory refresher course was due to the positive feedback from the participants and the improvement of test scores, as measured in our study. At this moment it is unclear whether every two years is the correct frequency to maintain the improved knowledge and keep up with the new developments in prenatal screening. Future studies could focus on repeating the questionnaire after a longer period of time to determine if knowledge is sustainable, to identify gaps of knowledge and to know how often the knowledge course is useful.

### Limitations

A limitation of this study is that knowledge of the counsellors was assessed in detail but it was not examined whether this knowledge has an impact on the quality of information provided to the pregnant women. Future studies could focus on this problem, as previous research only shows that low test score on knowledge from healthcare professionals coexists with low knowledge levels of parents^1^. No study has demonstrated that improvement of knowledge on prenatal screening improves the counselling quality. Although the majority of the counsellors (387/564 or 69%) answered to the T0 assessment questionnaires, a key limitation of this study is the low response rate on the T1 assessment of only 44% of the course attendees. The sample size is adequate enough to prove a 5–10% improvement in the test score. However, this study lacks generalisability, since it reflects only a certain group of counsellors. As the refresher course is mandatory every two years since 2017, we could verify our current findings during the next course since a larger and more generalizable cohort will attend. In addition, during this evaluation, it could be interesting to assess the counsellors’ attitude after the refresher course (at T1) as well. By doing this, we can see if the course itself, and the accompanying increase in knowledge, influences the attitude.

## CONCLUSIONS

A refresher counselling course is an effective way to improve knowledge of counsellors about prenatal screening, aneuploidies and structural anomalies. Health care professionals with clinical practice in sonography tend to have better baseline knowledge about prenatal screening and therefore may be more suitable for counselling. However, our study showed that a refresher course on counselling for prenatal screening improves knowledge of both counsellors with and without sonography experience. More importantly, after the refresher course there was no difference in scores between the two groups. In specific domains, counsellors’ knowledge needs to be improved to ensure that correct information is provided so that women and their partners can make informed decisions. Making the refresher counselling course mandatory is useful for the continuous improvement of healthcare practitioners’ knowledge. However, more research is necessary on the required frequency of a refresher course.
