# Supplementary material for: Effect of education and attitude on health professionals’ knowledge on prenatal screening
Source: Eur J Midwifery. 2020 Sep 22;4:38. doi: 10.18332/ejm/126626 (PMC7839104; doi:10.18332/ejm/126626)
Supplement: Supplementary file 1 [file EJM-4-38-s1.pdf]

Questionnaire

1	What is the purpose of prenatal screening on down syndrome and the fetal anomaly scan?<br>a) to achieve improvement of health<br>b) to inform future parents in time about the possibility of having a child with one or more disorders, so that they can make a choice concerning the treatment options<br>c) to lower the risks and costs of diagnostic investigations<br>d) to lower the costs of the care of children with a disability
2	Do patients have the right to refrain from receiving information about the possibilities of prenatal screening?<br>a) yes<br>b) no
3	Which of the following points about counselling the patient on prenatal screening should be noted in the patients file (multiple answers possible):<br>a) that counselling has taken place<br>b) the date of counselling<br>c) which screening tests the patient has chosen<br>d) if the patient objected against receiving information on trisomy 13 (patau syndrome) and trisomy 18 (edwards syndrome)
4	What does the sensitivity of a screening test indicate?<br>a) the chance that a affected person gets a positive test result<br>b) the chance that a healthy person gets a negative test result<br>c) the chance that, in case of a positive test result, a person is sick
5	What does the percentage false positives of a test indicate?<br>a) percentage of positive test results within the group with the illness<br>b) percentage of negative test results within the group with the illness<br>c) percentage of positive test results within the healthy group<br>d) percentage of negative test results within the healthy group
6	What indicates the positive predictive value of a screening test?<br>a) the chance that a sick person gets a justly unfavourable test result<br>b) the chance that a healthy person gets a justly good test result<br>c) the chance that the illness is justly diagnosed, if the test result is unfavourable
7	Most children with down syndrome are born to mothers younger than 36 years old

	a) true<br>b) false
8	The chance of conceiving a child with congenital heart disease is greater for women older than 36 years old<br>a) true<br>b) false
9	The most common type of down syndrome is not hereditary<br>a) true<br>b) false
10	Approximately one third of the pregnancies affected with down syndrome end with a miscarriage after the first trimester<br>a) true<br>b) false
11	Almost halve of the children with down syndrome are born with congenital heart disease<br>a) true<br>b) false
12	After introduction of the combined test (screening on trisomy 21, 13 and 18) in the Netherlands, the number of live births with down syndrome are more or less similar<br>a) true<br>b) false
13	What is in the Netherlands the prevalence of children born with down syndrome?<br>a) 1 in 400<br>b) 1 in 700<br>c) 1 in 1000
14	The combined test is less reliable on younger mothers compared to older mothers<br>a) true<br>b) false
15	The majority of pregnant women in the Netherlands choose the combined test<br>a) true<br>b) false
16	Women older than 36 years have a medical indication for invasive prenatal diagnostics<br>a) true

	b) false
17	Women older than 36 years have their combined test reimbursed<br>a) true<br>b) false
18	Down syndrome is associated with a reduced concentration of free beta hCG<br>a) true<br>b) false
19	Down syndrome is associated with a reduced concentration of PAPP-A<br>a) true<br>b) false
20	Slim pregnant women have on average higher concentrations of PAPP-A and free beta hCG than obese pregnant women<br>a) true<br>b) false
21	Most children with Edwards syndrome decease during the pregnancy or short after giving birth<br>a) true<br>b) false
22	Edwards syndrome is trisomy 13<br>a) true<br>b) false
23	Two pregnant women take the combined test. Woman nr. 1 has the following test result: 1:100. Woman nr. 2 has a test result of 1:200. The chance of a child with down syndrome is higher for woman nr. 2<br>a) true<br>b) false
24	A pregnant woman takes the combined test and receives as test result a chance of 1 in 10 on down syndrome. The chance that this woman has a fetus with down syndrome is higher than the chance of a fetus with a normal chromosomal array<br>a) true<br>b) false
25	As a prenatal centre, you’ve started offering the combined test. You offer a chorionic villous sampling if the chance is 1 on 200 or higher. Until now 150 chorionic villous

	samplings have been performed without a positive test result for down syndrome. These results show that the quality of the combined test in your centre is insufficient<br>a) true<br>b) false
26	If at 12 weeks of gestation the nuchal translucency thickness is greater than 3,5 mm but the karyotype is normal, parents can be reassured<br>a) true<br>b) false
27	Monochorionic pregnancies are always monozygotic<br>a) true<br>b) false
28	In case of a twin pregnancy with one abnormal fetus and one healthy fetus, the twin pregnancy can be monozygotic<br>a) true<br>b) false
29	If the pregnant woman will never consider termination of pregnancy, it is not necessary to discuss the prenatal screening options<br>a) true<br>b) false
30	It is not allowed to put information about prenatal screening on your website, because some parents do not want to receive information<br>a) true<br>b) false
31	What is the purpose of the fetal anomaly scan?<br>a) to detect down syndrome<br>b) to detect neural tube defects<br>c) to reassure the pregnant woman
32	What is the definition of a late pregnancy termination?<br>a) pregnancy termination after the first trimester<br>b) intra-uterine fetal demise after 20 weeks of gestation<br>c) pregnancy termination after 24 weeks of gestation
33	What is the prevalence of spina bifida in the Netherlands?<br>a) 1 in 200 pregnancies = circa 900/year

	b) 1 in 2000 pregnancies = circa 90/year<br>c) 1 in 20000 pregnancies = circa 9/year
34	The youngest child from a previous marriage of the father has spina bifida. Does this influence your planning of the fetal anomaly scan?<br>a) no, I will schedule the fetal anomaly scan as normal<br>b) yes, I will schedule the fetal anomaly scan earlier than normal<br>c) yes, I will schedule an advanced fetal anomaly scan instead of a regular fetal anomaly scan<br>d) no, I will schedule the fetal anomaly scan as normal and will also schedule an advanced fetal anomaly scan
35	Imagine, you receive the results of a fetal anomaly scan where not all organ systems are reviewed. What do you do?<br>a) I will request a repeat fetal anomaly scan<br>b) I will ask the parents if they want to carry out a follow-up scan<br>c) I will reassure the parents<br>d) I will refer parents for an advanced fetal anomaly scan
36	What is the prevalence of severe congenital heart disease in the Netherlands?<br>a) 1 in 125 = circa 1400 newly born per year<br>b) 1 in 250 = circa 700 newly born per year<br>c) 1 in 500 = circa 350 newly born per year
37	Prenatal diagnosis of congenital heart disease can improve the prognosis in some cases<br>a) true<br>b) false
38	The NIPT is to determine the health of the unborn baby<br>a) true<br>b) false
39	If the result of the NIPT shows trisomy 21, there is still a small chance that there is no chromosomal anomaly<br>a) true<br>b) false
40	In case of a normal NIPT, there is still a small chance on a child with trisomy 21, 18 or 13<br>a) true

	b) false
41	If the NIPT result is abnormal, the pregnancy can be terminated without further tests<br>a) true<br>b) false
42	Women ≥ 36 years old have a medical indication for NIPT<br>a) true<br>b) false
43	In the Netherlands the NIPT is performed at 10 weeks of gestation age or more<br>a) true<br>b) false
44	The NIPT is not successful in 1 to 3 on 100 women (1-3%)<br>a) true<br>b) false
45	If pregnant women have had a child with down syndrome, they must take the combined test before they can apply for the NIPT test<br>a) true<br>b) false
46	Which test tracks down the most foetuses with down syndrome?<br>a) the combined test as first screenings test<br>b) first the combined test and then the NIPT<br>c) NIPT as first screenings test
47	Which test path has the least amniocenteses because the test result shows a high chance on down syndrome?<br>a) combined test<br>b) combined test an then the NIPT<br>c) NIPT
48	Chorionic villous sampling is only performed at 11 weeks of gestation or more<br>a) true<br>b) false
49	Amniocentesis is only performed at 12 weeks of gestation or more<br>a) true<br>b) false

50	Amniocentesis and chorionic villous sampling are more certain tests for trisomy than the NIPT<br>a) true<br>b) false
51	The counsellor has the primary responsibility for setting the indication for an advanced fetal anomaly scan<br>a) true<br>b) false
52	The ultra-sonographer has the responsibility to verify before every fetal anomaly scan if the woman has an indication for an advanced fetal anomaly scan<br>a) true<br>b) false
53	In case there is an indication for an advanced fetal anomaly scan but the woman chooses to have the regular fetal anomaly scan, the counsellor has to inform the woman that referring the woman for a regular fetal anomaly scan is incorrect and culpable<br>a) true<br>b) false
54	In case of incorrect referral of a woman who has an indication for an advanced fetal anomaly scan but is referred for an regular anomaly scan, both the referring party as the ultra-sonographer can encounter juridical and disciplinary problems<br>a) true<br>b) false
55	The fetal anomaly scan is for screening for structural anomalies of the unborn baby and examine for pregnancy complications that can endanger the health of the baby<br>a) true<br>b) false

© 2020 Jansen C. H. et al.
